# Collaborative Mining and Interpretation of Large-Scale Data for Biomedical Research Insights

**DOI:** 10.1371/journal.pone.0108600

**Published:** 2014-09-30

**Authors:** Georgia Tsiliki, Nikos Karacapilidis, Spyros Christodoulou, Manolis Tzagarakis

**Affiliations:** 1 School of Chemical Engineering, National Technical University of Athens, Athens, Greece; 2 University of Patras and Computer Technology Institute & Press ‘Diophantus’, Patras, Greece; Children's Medical Research Institute, Australia

## Abstract

Biomedical research becomes increasingly interdisciplinary and collaborative in nature. Researchers need to efficiently and effectively collaborate and make decisions by meaningfully assembling, mining and analyzing available large-scale volumes of complex multi-faceted data residing in different sources. In line with related research directives revealing that, in spite of the recent advances in data mining and computational analysis, humans can easily detect patterns which computer algorithms may have difficulty in finding, this paper reports on the practical use of an innovative web-based collaboration support platform in a biomedical research context. Arguing that dealing with data-intensive and cognitively complex settings is not a technical problem alone, the proposed platform adopts a hybrid approach that builds on the synergy between machine and human intelligence to facilitate the underlying sense-making and decision making processes. User experience shows that the platform enables more informed and quicker decisions, by displaying the aggregated information according to their needs, while also exploiting the associated human intelligence.

## Introduction

Biomedical research is nowadays associated with large-scale, ever-increasing amounts of multiple types of data, obtained from diverse and distributed sources. A vast growth of publicly available biomedical resources, including multiple types of data sets and analysis tools, are available on the web. Researchers have the advantage to access complementary views of a single organism by analyzing multiple types of data, including whole genome sequencing, expression profiling and other high-throughput experiments [1]. Recent technology advances, such as those in Next Generation Sequencing (NGS) platforms, entail an exponential increase in the size and number of experimental data sets available [2].

Biomedical research has been revolutionized by this data explosion [3], whilst becoming increasingly interdisciplinary and collaborative in nature [4], [5]. In such settings, data may vary in terms of subjectivity and importance, ranging from individual opinions and estimations to broadly accepted practices and well-documented scientific results. Data types can be of diverse level as far as human understanding and machine interpretation are concerned. Researchers face difficulties when they have to consider and exploit accumulated data, and meaningfully analyze them towards making a decision [6]. Under a typical working scenario, researchers need to aggregate big volumes of data from multiple sources, and then analyze them for insights that would very unlikely emerge from manual inspection or analysis of any single data source [7]. This would require support to various levels of engagement with those data, without necessarily requiring deep comprehension of database functionalities [8].

The above remarks advocate the exploitation of the synergy between human and machine reasoning when designing systems to support such collaboration and decision making activities [9]. Exploitation of data mining technologies for pattern and dependencies discovery within large data sets is certainly of great benefit. However, in spite of big progress made in the area of computational analysis, there are many patterns that humans can easily detect but computer algorithms struggle to estimate [10]. Additionally, interpretation of analysis results is a challenging issue here; besides of getting results from the execution of a statistical algorithm, additional information is needed concerning data input format as well as the statistical model's assumptions or parameters. Maintenance of this data provenance through appropriate metadata would enable researchers to repeat experiments with alternative assumptions or data sets [11].

This paper reports on the practical use of an innovative web-based collaboration support platform in a biomedical research context, which is in line with the above requirements and has been developed in the context of the Dicode EU FP7 research project (http://dicode-project.eu/). The Dicode solution is generic, in that it is able to address collaboration and decision making needs of diverse contexts. Beyond the biomedical research context, its applicability has been also tested in medical treatment decision making and in opinion mining Web 2.0 data. The proposed solution adopts an integrated approach that facilitates the identification, assembly and analysis of big multi-faceted data. Moreover, it fully embeds data mining in a collaborative data analysis and decision making process. The above are performed through a meaningful integration of collaboration, decision making and data mining services that enable users to:

- share their own data, models, experiences and findings;
- efficiently handle large amounts of data and avoid out-of-memory errors;
- trigger and exploit a set of mining algorithms that are tailored to biomedical research needs;
- integrate heterogeneous clinico-genomic data sources with advanced analytical techniques;
- share and collaboratively interpret the outcomes of the above mining algorithms;
- consider alternative visualizations for the process of the underlying collaboration;
- monitor data and decision provenance issues.

The remainder of the paper comments on related work, shows details about the overall approach followed in the Dicode project, and describes an illustrative scenario to demonstrate the use of the proposed platform in the biomedical research context. Evaluation results show that the platform enables users to make better, more informed and quicker decisions. Concluding remarks are discussed in the last section of the paper.

## Related work

The emergence of the Web 2.0 era introduced a plethora of collaboration tools which enable massive scale engagement and feature novel paradigms. For instance, Thinkature (http://thinkature.com/) permits the representation of ideas and concepts that can be interconnected to form meaningful diagrams, DropBox (https://www.dropbox.com/) is extensively used for file sharing, ActiveCollab (http://www.activecollab.com/) for project management, Cohere (http://cohere.open.ac.uk/) for argumentative collaboration, and GitHub (https://github.com) for software development and collaboration. These tools cover a broad spectrum of needs. However, they are generic and - in most cases - very difficult to interoperate; thus, their separate use becomes cumbersome and time consuming.

Focusing on the biomedical research domain, a number of projects and initiatives aim at addressing diverse collaboration requirements in a variety of contexts. For instance, GRANATUM (http://granatum.org) attempts to bridge the information, knowledge and collaboration gap by providing integrated access to the globally available data resources needed to perform complex cancer chemoprevention experiments and conduct studies on large-scale datasets; Health-e-Child (http://www.health-e-child.org) offers clinicians a comprehensive view of a child's health by integrating biomedical data, information and knowledge that spans the entire spectrum from imaging to genetic, clinical and epidemiological data; Virolab (http://www.virolab.org) offers a user friendly environment to facilitate tasks such as data archiving, data integration, data mining and simulation; finally, SIMBioMS (http://simbioms.org) is a multi-module solution for biomedical data management that is able to accommodate experiments requiring non-conventional data storage solutions. Although the above projects are addressing specific biomedical subjects, they do not deal with big data issues; also, they do not exploit the synergy between human and machine intelligence in order to meaningfully accommodate and interpret the results of the associated data mining services through an environment that facilitates and enhances collaboration. Along these lines, a noteworthy initiative by the american National Institutes of Health is called Big Data to Knowledge (BD2K; http://bd2k.nih.gov/) which aims to develop the new approaches and tools that will enhance the use of biomedical ‘Big Data’ by supporting research, implementation, and training in data science and other relevant fields.

Many applications and web services that link together bioinformatic tools and databases have recently emerged, showing the way to easily visualize and analyze biomedical data. For instance, BioGRID [12], BNDB [13] and BioMart [14] are repositories which store readily combined data sets and provide platforms to easily visualize such data. Oncomine [15] and SubMap [16] are associating data integration and meta-analysis. The GenePattern platform provides access to more than 180 tools for genomic analysis to enable reproducible in silico research (http://www.broadinstitute.org/cancer/software/genepattern/). In addition, many collaborative resource sharing networks have been established, e.g. the eagle-i consortium (https://www.eagle-i.net/), to address the data sharing needs and accelerate the discovery of new knowledge amongst researchers. Integration of these separate systems and resources into a single infrastructure that streamlines heterogeneous workloads is a challenging task. Two examples of research computer systems for data integration are caBIG and BIRN's cyber infrastructure. The Cancer Biomedical Informatics Grid (caBIG) is a network to enable sharing of data and software tools across individuals and cancer research institutions to improve the pace of innovations in cancer prevention and treatment (http://cabig.cancer.gov). The Biomedical Informatics Research Network (BIRN) is a distributed virtual community of shared resources that currently supports the sharing and analysis of neuroimaging data (http://www.nbirn.net).

As the number of related Web services is constantly increasing, their proper integration becomes critical. Aiming to address this issue, myExperiment [17] offers an online environment that supports the social sharing of bioinformatics workflows, i.e. procedures consisting of a series of computational tasks, which can then be reused according to their specific requirements. Another representative example in this category of tools is BioCatalogue (http://www.biocatalogue.org/), which is a registry of web services that allows users to annotate and comment on the available services in order to assist them in identifying the more suitable ones (services are presented in terms of their functions, data types and resources). A third example is MethodBox (https://www.methodbox.org/), which enables researchers to browse and download data sets, share methods and scripts, find fellow researchers with similar interests and share knowledge. Instead of workflows, MethodBox users share statistical methods for epidemiology and public health research. Finally, the Galaxy Project (http://galaxy.psu.edu/) offers a web-based platform that allows researchers to perform and share their analyses. In any case, approaches of this category demonstrate a set of limitations, mainly concerning incorporation of collective intelligence and flexibility in the integration of services offered. Moreover, they lack mechanisms for a meaningful integration of data mining services to appropriately support tasks such as the discovery of patterns and dependencies within large data sets, which are very common in the biomedical research domain.

## The Dicode approach

The overall goal of the Dicode project was to facilitate and augment collaboration and decision making in diverse data-intensive and cognitively-complex settings. To do so, whenever appropriate, it builds on prominent high-performance computing paradigms and large scale data processing technologies to meaningfully search, analyze and aggregate data existing in diverse, extremely large, and rapidly evolving sources. At the same time, particular emphasis was given to the proper exploitation and analysis of large scale data (considering the associated issues of volume, variety, velocity and value), as well as to collaboration and sense making support issues. It enables the meaningful incorporation and orchestration of a set of interoperable web services that reduce the data-intensiveness and complexity overload of the settings under consideration to a manageable level, thus permitting users to be more productive and effective in their work practices. Dicode services cover a variety of data acquisition, data mining, collaboration support, and decision making support needs [18].

### The Dicode Workbench

The above mentioned interoperation of the Dicode services is performed through the Dicode Workbench, a web-based application that enables the integration of heterogeneous services and ensures their interoperability from both a technical and a conceptual point of view. Semantics techniques have been exploited to define an ontological framework for capturing and representing the diverse stakeholder and services perspectives.

**Figure 1:**
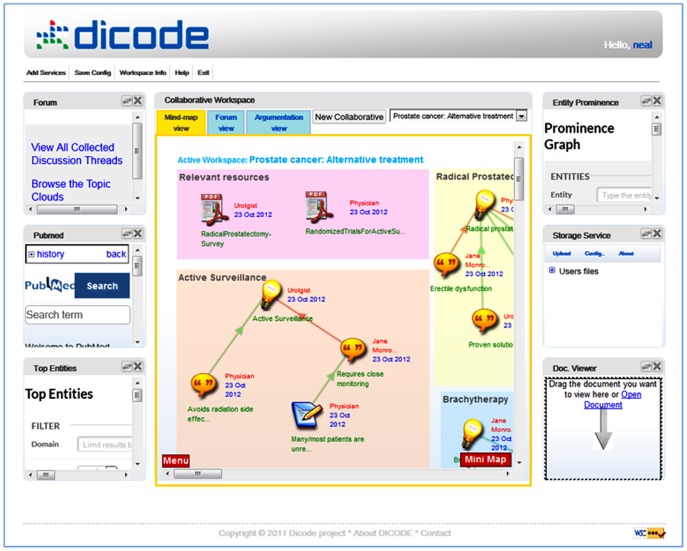
An instance of the Dicode Workbench.

Figure 1 illustrates an instance of the Dicode Workbench. As shown, a widget-like approach [19] has been adopted, where each widget implements a particular web service (i.e. services developed either inside or outside the context of Dicode). In other words, Dicode components are wrapped into services and integration is performed on a service level. This approach does not pose any restrictions on the back-end technology and programming language used for the development of each service (for instance, the Collaboration Support Services discussed in the next subsection are implemented in C#, while the Subgroup Discovery Service discussed in the subsection ‘Context-related Dicode Services’ is implemented in C++). The Dicode Workbench has been implemented using Java technologies, i.e. JavaServer Pages (JSPs) and Servlets [20]. It is publicly available at http://hodgkin.dia.fi.upm.es:8080/dicode. The widget toolkit exploited in Dicode was the Google Web Toolkit (GWT) [21], which is based on Java and provides a set of core Java APIs and Web widgets.

The Workbench can be personalized, in the sense that an end user may add or remove widgets (for example, according to the needs of the particular context and issue under exploration). The central widget of Figure 1 hosts the Collaboration Support services (which are further analyzed in the next section), while widgets on the right and left side host various data acquisition and data mining services. The Dicode Workbench allows users to maximize any of the widgets located on the sides; if prompted to do so, the selected widget moves to the center of the window to reflect the current focus of the attention.

Technically speaking, the Dicode Workbench uses *iframe* elements to display the services (each iframe hosts a particular service). The service displayed in the iframe may use any of the state-of-the-art web technologies such as HTML5, CSS3, JavaScript, AJAX or jQuery. To integrate a service in the Dicode Workbench, service providers have to follow a number of necessary steps: develop the service (including the implementation of the service logic and the necessary public interface for invoking the service - usually, the exchange of structured information is based on RESTful calls or WS-* (SOAP) [22]), develop the web interface of the service (to allow user interaction with the service), deploy the service and the web interface (both accessible through an URL/URI to the web server hosting the service), and finally register/publish the service in the Dicode Registry of Services (DRS). DRS is an integrated component of the Dicode Workbench that maintains the necessary information for each Dicode service (i.e. useful metadata and annotations contained in the Dicode ONtology (DON) [23], the URI and the provider of the service, a description of its functionality, comments from users). Through DRS, users are aware of the available services and their use. Moreover, DRS maintains information concerning a service's metrics (such as times used, successful attempts, and average/minimum/maximum times of execution).

Beyond integration at the level of the user interface, Dicode services are also integrated at a deeper, semantic level. This type of integration allows services to exchange data for a particular purpose (this is described in detail below, in the context of the Dicode Collaboration Support Services and the particular scenario of use). It also supports user friendly functionalities, such as ‘drag-and-drop’ for passing of either input or output data from one service to another. Data exchange among Dicode services is possible through a loosely coupled architecture that is built upon the idea of message passing interfaces (MPI) following a ‘publish-subscribe’ design pattern [24]. In particular, we focused on the *postMessage* mechanism provided by HTML5 (http://dev.w3.org/html5/postmsg/). This mechanism allows applications running in different windows to communicate information across various origins and domains. A detailed technical description of diverse integration issues in Dicode appears in [25] and [26].

### The Dicode Collaboration Support services

Being fully integrated into the Dicode Workbench, Collaboration Support services enable participants to collectively reflect on various issues, with their ultimate aim being to jointly decide about which course of action to take. They facilitate the synchronous and asynchronous collaboration of stakeholders through adaptive workspaces, efficiently handle the representation and visualization of the outcomes of the data mining services (through alternative and dedicated data visualization schemas) and create workflows. In addition, these services provide an interactive search and analysis mechanism for indexing and searching of standard documents.

Collaboration in Dicode brings together two paradigms: the Web 2.0 paradigm, which builds on flexible rules favoring ease-of-use and human interpretable semantics, and the traditional decision support paradigm, which requires rigid rules that reduce ease-of-use but render machine interpretable semantics. To achieve this, our approach builds on a conceptual framework, where formality and the level of knowledge structuring during collaboration is not considered as a predefined property, but rather as an adaptable aspect that can be modified to meet the needs of the tasks at hand. By the term formality, we refer to the rules enforced by the system, with which all user actions must comply. Allowing formality to vary within the collaboration space, *incremental formalization*, i.e. a stepwise and controlled evolution from a mere collection of individual ideas and resources to the production of highly contextualized and interrelated knowledge artifacts can be achieved [27].

Dicode offers alternative visualizations of the collaboration space (called *Dicode views*), which comply with the incremental formalization concept. Each Dicode view provides the necessary mechanisms to support a particular level of formality. The more informal a view is, the greater easiness-of-use is implied. At the same time, the actions that users may perform are intuitive and not time consuming; however, the overall context is human (and not system) interpretable. On the other hand, the more formal a view is, the smaller easiness-of-use is rendered; the actions permitted are less and less intuitive and more time consuming. The overall context in this case is both human and system interpretable [28].

**Figure 2:**
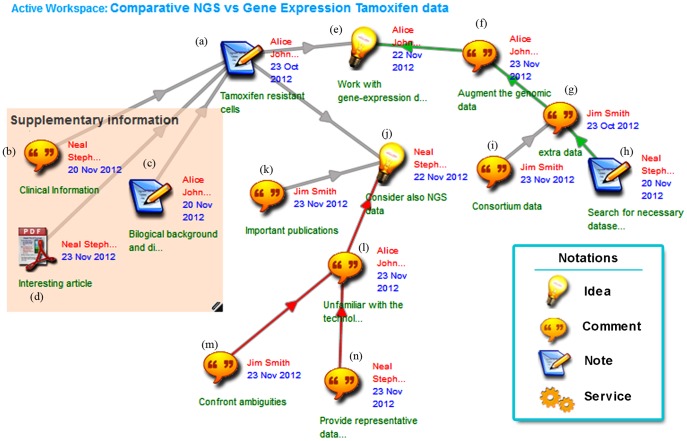
Launching a collaboration workspace for estimating the dominance of Tamoxifen resistant cells to global gene expression. Alice and her colleagues upload and link related biological, clinical or technical information. Notations used in Dicode workspaces are shown in the bottom right box.

The functionality described in the next section of this paper is offered through the Dicode *mind-map view*, in which a collaboration space is displayed as a mind map (Figure 2), where users can upload and interrelate diverse types of items. This view deploys a spatial metaphor permitting the easy movement and arrangement of items on the collaboration space. The aim of this view is to support *information triage* [29], i.e. the process of sorting and organizing through numerous relevant materials.

In the ‘mind-map view’ of the collaboration space, stakeholders may organize their collaboration through dedicated item types such as ‘ideas’, ‘notes’, ‘comments’ and ‘services’. Ideas stand for items that need further exploitation; they may correspond to an alternative solution to the issue under consideration and they usually trigger the evolution of the collaboration. Notes are items expressing one's knowledge about the overall issue, an already asserted idea or note. Comments are items that usually express less strong statements and are uploaded to express some explanatory text or point to some potentially useful information. Finally, service items enable the interoperation with and exploitation of external services; they permit users to configure, trigger and monitor the execution of web services from within a Dicode workspace, and allow the automatic upload of their results into it (as soon as the execution of the service is completed). Configuration and triggering of a service is performed through dedicated web interfaces, developed by the corresponding service's provider, which convey the neccessary parameters for the execution of the service. Multimedia resources can also be uploaded into the mind-map view (the content of which can be displayed upon request or can be directly embedded in the workspace).

## A Biomedical Research Assimilator Context

The work presented here concerns multidisciplinary biomedical research communities, ranging from biologists to bioinformaticians, which need to collaborate in order to assimilate clinico-genomic research information and scientific findings, as well as explore diverse associated issues. In many cases, such collaboration is based on the outcome of large scale data analysis. Under this context, certain difficulties arise in terms of accessing, storing, processing and interpreting results based on genomic and clinico-genomic data, which points to the need for every scientist to understand how to manage, navigate, and curate large-scale data [30], [31].

Dicode is able to fully serve the requirements of a typical working scenario in the biomedical domain, in order to built a useful pipeline for the analysis of genomics and transcriptomics data [32]. A typical process would be to download the raw or pre-processed data from a database (e.g. Gene Expression Omnibus, GEO) along with all the relevant phenotypical and clinical information needed to understand and analyze the data. That could result in augmenting the already available in-house data with publicly available data stored in varying formats. An intermediate but important step would be to reformat and store them locally, in order to visualize and analyze them. The analysis could be conducted by using either a standalone tool, such as Cytoscape (http://www.cytoscape.org/), or in-house scripting using, for instance, the R statistical language (http://www.r-project.org/).

Perhaps the most important step in the life cycle of an experiment is to interpret and communicate the findings. The results need to be comparatively assessed against modern methodologies; most importantly, they need to be biologically or medically interpreted to have an insight into the initial question of interest. For that purpose, researchers confer with databases, such as the Kyoto Encyclopaedia of Genes and Genomes, or standalone tools which are directly linked to databases and can qualitatively and quantitatively assess the submitted results using the database resources (for example, Cytoscape mentioned above).

Table 1 provides information for publicly available genomics and transcriptomics data that can be incorporated in the settings under consideration. These data are related to breast-cancer disease, but it could be easily generalized to other diseases (e.g. cardiovascular disease) or organisms (e.g. plant data). To give an indication of the data scale associated to the context under consideration, representative numbers of samples and data sizes are given.

**Table 1:** Input data considered for the biomedical research assimilator context.

Data type & description	Databases (web available)	Data in numbers
**Genomics/Transcriptomics data: Normalized or raw data**	Gene Expression Omnibus	86 datasets, 7, 607 samples (∼500 Kb per sample, ∼32 Mb per dataset)
	ArrayExpress	987 experiments; 69, 483 samples
	Stanford Microarray Database	508 experiments
**Phenotypic data: Supplementary clinical or phenotypic data available**	As above	2 files on average per dataset (∼10 Kb per dataset)
**Molecular Pathways: Data from known and established molecular networks**	Kyoto Encyclopedia of Genes and Genomics (KEGG)	416 pathway maps (153, 758 total)
	Reactome	3, 931, 211 data entries
**Annotation data: Reference databases for biomedical & genomic information**	Gene Ontology (GO)	∼30, 000 terms, ∼50, 000 relationships
	National Center of Biotechnology Information	26, 473 annotated coding regions (RefSeq), 129, 493 homo sapiens entries (Unigene), ∼127 billion bases (GenBank), >21 million citations for biomedical literature (PubMed)

### Context-related Dicode Services

For clinico-genomics research, the Dicode Workbench is the integration platform for accessing and assessing available resources and tools through an interface that bundles all functionalities together. It is the integration platform for all Dicode data analysis and support services. The *Storage service*, built to comfort the sharing and exchange of information (files, reports, etc.) in data-intensive and cognitively-complex settings, is embedded within the Workbench. This service provides all functionalities needed to allow permanent and reliable storing of files as well as their accessibility. Other Dicode services exploited in the specific context are:

- The *Collaboration Support services* (described above), which exploit the reasoning abilities of humans to facilitate sense-making of the Dicode data mining services' results and capitalize on their outcomes.
- The *Decision Making Support services*, which exploit machine-interpretable data and semantics to enhance individual and group decision-making. This is performed through dedicated views of a workspace that support stakeholders in arguing about the issue under consideration, whilst providing them with appropriate notifications and recommendations given their preferences, competences, expertise etc. A detailed description of these services appears in [33].
- The *Forum Summarization service*, which receives clusters of discussion threads as input from relevant public forums and identifies their most prominent terms (topics). The identified topics can be used to derive the main theme in the cluster supplied.
- The *Subgroup Discovery service*, which searches for subgroups in any user provided data by searching the rules that cover target and non-target value examples [34]. Particularly, this service finds patterns in the data which are highly associated with a variable of interest. It supports two different subgroup discovery data mining algorithms.
- The *Recommendation service*, which recommends similar users or documents from log file data based on similarity models learned by using the Dicode *Similarity Learning Service* [35]. Specifically for the biomedical research assimilator context, the GEO-Recommender (GEOR) web-based application is employed to search the GEO database for appropriate datasets based on keywords or the description supplied by the user.
- The *PubMed service*, which searches for relevant (to the topic of discussion) scientific articles from the PubMed database (http://www.ncbi.nlm.nih.gov/pubmed).

Additional data analysis applications can be easily uploaded into the Dicode Workbench, provided that they are wrapped in a web service (see section ‘The Dicode Workbench’). This offers a great opportunity for researchers to upload their own code and collaborate with their peers in improving it, or discussing their findings by using one of the above mentioned Dicode services. In any case, the input and output files formats are tab-delimited text files (txt) and comma separated files (csv), which allows their exchangability whenever possible.

Descriptive statistics are also calculated for each Dicode data mining service called, i.e. overall sample size, sample size per class (e.g. treatment or exposure), mean and variance per class, minimum and maximum value per class. Depending on the Dicode service called, those descriptive statistics are calculated for the output of the method. For instance, for the Subgroup Discovery service, the above mentioned statistics are calculated for each subgroup estimated by the algorithm.

### Scenario of use

To better demonstrate the use of the proposed web-based collaboration support platform, this subsection presents an illustrative scenario concerning collaboration in the area of breast cancer research (a recording of the platform's use appears at http://dicodedev.cti.gr/screencast/screencast.html - to view it, Adobe Flash Player is required). Here we emphasize how the Dicode Collaboration Support Services can be used within an integration framework in order to support data mining and decision making tasks.

Alice is a Pharmacology Ph.D. student. Her research is on adjuvant hormonal therapy for patients with breast cancer disease; particularly, she is interested in identifying how Tamoxifen (Tam) resistant cells modulate global gene expression. Tam is a widely used antagonist of the estrogen receptor (ER), whereas its resistance is a well-known obstacle to successful breast cancer treatment [36]. While adjuvant therapy with Tam has been shown to significantly decrease the rate of disease recurrence and mortality, recurrent disease occurs in one third of patients treated with Tam within five years of therapy. Alice selected and analyzed gene-expression data from 300 patient samples with the help of Neal, an MD at a collaborating university hospital, and Jim, a postdoctoral researcher in Bioinformatics. These data are derived from whole human genome expression arrays (Affy U133A Plus 2.0 see http://www.affymetrix.com). Although the sample is relatively large, Alice believes that augmenting the data with publicly available data will be a good idea for statistically significant results.

To analyze the data and discuss the analysis results, Alice, Neal and Jim decide to collaborate by using the Dicode mind-map view. In this direction, Alice is launching a new collaborative workspace (Figure 2). Even though all three collaborators are aware of the benefits and difficulties of Tam treatment, Alice adds a note on the collaboration workspace to fully explain the characteristics of the genomic data (Figure 2, (a)). Neal has collected all the necessary clinical information and posts them on the collaboration space (Figure 2, (b)). Apart from stating its background and technical difficulties (Figure 2, (c)), Neal finds an interesting article concerning the Tam treatment and uploads the corresponding pdf file on the workspace (Figure 2, (d)). In the mind-map view, users may group together related items by using coloured rectangles (see, for instance, the one entitled Supplementary information, which was drawn by Neal).

Alice believes that they should first analyze the gene-expression data (idea item (e), Figure 2) that they should later augment (comment item (f), Figure 2). Jim suggests launching the GEORecommender (GEOR) service (Figure 2, (g)) to find ‘similar’ data sets in terms of pathology characteristics. GEOR is a web service implemented in Dicode, which searches the GEO database (http://www.ncbi.nlm.nih.gov/geo/) based on keywords or the description supplied by the user. Having that in mind, Neal offers to find the extra data sets (Figure 2, (h)), since he is more confident with the technical characteristics of the data. Jim agrees (Figure 2, (i)), and adds that there are data available from consortiums such as caBIG, which have extensively proven the need to augment or at least compare and assess findings across multiple data sets.

Even though Alice believes that they should first work with the gene-expression data, Neal argues that they should also consider NGS data (idea item (j), Figure 2). He mentions that he is responsible for a clinical trial and can have access to total RNA from human breast cancer cell lines, which are then analyzed using NGS technology. Jim is also working with NGS data and he is highly recommending the integration or at least the comparative study of the two platforms. He has recently published some important results (Figure 2, (k)) by classifying publicly available transcriptomics data and he has found striking similarities between the two. Moreover, NGS is the latest technology having higher specificity and sensitivity, and thus has higher potential in meaningfully augmenting Alice's results.

Alice is reluctant to start working with NGS data because she is unfamiliar with the technology and argues that she will probably invest time without being assured about the significance of the results (Figure 2, (l) note that arrows in red denote argumentation against the ‘parent’ item, while arrows in green denote argumentation in favor). To defeat this statement, Neal suggests (Figure 2, (m)) to upload a representative data set from his laboratory, while Jim offers to help her (Figure 2, (n)) deal with all the annoational ambiguities between the two datasets.

Alice thinks about exploiting the Subgroup Discovery (SD) data mining algorithm [37] [34]. SD estimates patterns in the data (‘subsets’) which are highly correlated with a target attribute. This is a popular approach for identifying interesting patterns in the data, since it combines a sound statistical methodology with an understandable representation of patterns. For example, in a group of patients that did or did not respond to specific treatment, an interesting subgroup would consist of patients who are older than 60 years and do not suffer from high blood pressure and succesfully respond to the treatment (compared to the average response).

**Figure 3:**
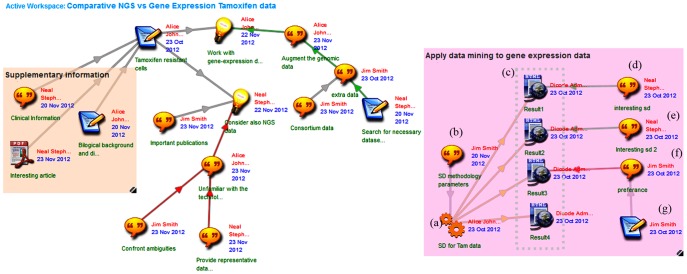
Application of SubGroup Discovery service to gene expression data and assessment of results.

To invoke the SD algorithm, Alice uploads the associated service item on the workspace (Figure 3 (a)) and follows the necessary configuration steps to start the execution of the service. Configuration includes the specification of the URI for the REST-based SD service and specification of parameters such as input file, number of rules to be used, service ontology, and minimum number of subgroups to be retrieved. Jim advises her on the SD methodology parameters (Figure 3, (b)); particularly, they decide to run the algorithm with a minimum number of four subgroups for each biological category to emphasize only the highly ranked statistically significant groups of the data. Once they have made up their decision concerning the input parameters of the SD service, Alice triggers its execution.

Upon the successful termination of the service, the output is automatically uploaded on the collaboration workspace (Figure 3, (c)). Collaboration items are created for each estimated subgroup (in this scenario, output is given in html format), and particularly, they are tables of GO (http://www.geneontology.org/) and KEGG (http://www.genome.jp/kegg/) terms, which describe biological processes related to the estimated groups of genes. For this particular run, the SD results are summarized in the following four subgroups: ‘sequence specific DNA binding TFA’, ‘transcription from RNA polymerase II promoter’, ‘signaling transducer activity’, ‘PI3 k-Akt signaling’ (Results 1–4, Figure 3, (c)).

The results of the SD service seem convincing to Neal (Figure 3 (d–e)), while Jim expresses his opposition about the third outcome and quotes a part of a scientific paper he recently read (Figure 3, (f–g)). Note that in terms of data integration, GO database information can be of great assistance when used as input for any Gene Sets Enrichment Analysis tool [38], which besides interpreting gene expression data, it is also widely applied to match patterns identified amongst various -omics data [39] [40]. Dicode offers a service to map gene, probe or protein ids to GO ids [34], thus offering the option to compare results amongst diverse data sets.

**Figure 4:**
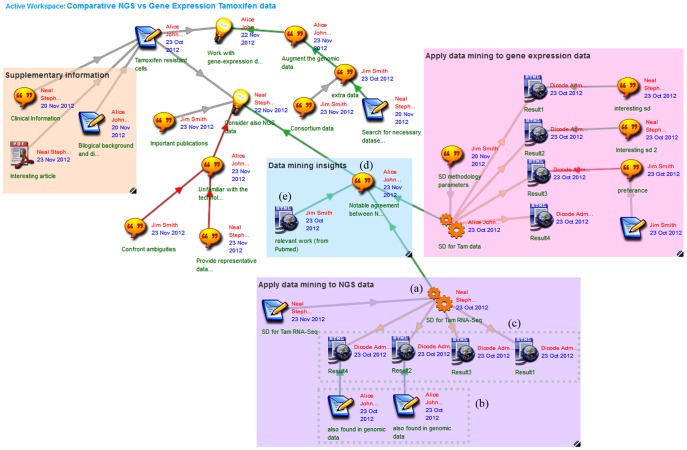
Application of SubGroup Discovery service to next generation sequencing data, assessment of results, and insights.

The same procedure (invoking the SD service and collectively assessing its output) is followed for the NGS data (Figure 4, (a–b)). The three researchers carefully examine the commonalities between the two SD runs (on genomic and NGS data) and share their insights. The subgroups returned for the NGS data ((Figure 4, (c)) are very similar to the ones obtained from SD service on genomic data (Results 1–4 correspond to: ‘response to stimulus’, ‘positive regulation of transcription’, ‘transcription from RNA polymerase II promoter’, ‘signaling transducer activity’). Alice is impressed with the commonalities found between the two SD runs; she is now convinced that there is scope to integrate additional NGS data. She expresses her insight (Figure 4, (d)) and links it to the original Neal's idea (note that SD service items are also linked as arguments in favor of this insight). To further elaborate this issue, Jim uses the PubMed service offered through the Dicode Workbench to search for recent relevant articles. He then uploads a link (Figure 4, (e)) pointing to a scientific report that strengthens Alice's argument.

The above collaboration may proceed to further augment the gene expression and NGS data. For instance, as Jim has previously suggested, the researchers involved may invoke GEOR to continue the analysis with the data sets that Neal has already downloaded.

## Evaluation

The Dicode platform has been already introduced in three real-life settings (i.e. the biomedical research assimilator, decision making on clinical treatment effects, and opinion mining from unstructured Web 2.0 data) for a series of pilot experimentations. For the clinico-genomics research assimilator context 61 users from four European institutes participated in a detailed evaluation of the platform, concerning both individual Dicode services and the Dicode Workbench. Users had a varying level of hands-on experience in related technologies (ranging from ‘early adopters’ to semi-experienced and novice users); their background was on disciplines such as Bioinformatics, Biology and Computer Science. No consent was given because data were analyzed anonymously. The suggested framework involves research conducted in established educational settings, therefore it was exempted from being reviewed and approved by an institutional review board [41]. The above decision was made by the ethics committee of the Dicode project, designated to oversee all research ethics matters concerning research conducted by all project's partners.

Feedback requested was of both quantitative and qualitative type. Answers to the quantitative questions of the questionnaires were given for ordinal data in a 1–5 scale (questions concerning the quality, acceptability and accessibility of the services provided), where 1 stands for ‘I strongly disagree’ and 5 for ‘I strongly agree’, and for continuous numerical data (scale data) in a 0–10 scale (questions concerning the services' usability), where 0 stands for ‘none’ and 10 for ‘excellent’ [42], [43].

As far as the overall quality of the Dicode Collaboration Support services is concerned (Table 2), the evaluators agreed that: the objectives of the services are met (median = 4, mode = 3), the services are novel to their knowledge (median = 4, mode = 4), they are satisfied with the performance of the services (median = 4, mode = 4), and they are overall satisfied with these services (median = 4, mode = 4). The evaluators seemed to be to some extent sceptical as to whether the services are able to address the data intensive decision making issues (median = 3, mode = 3).

**Table 2:** Overall Quality Descriptive Statistics for the Dicode Collaboration Support Service.

Question	Median (scale 1–5)	Mode (scale 1–5)
Q1: The service is able to address data intensive decision making issues	3	3
Evaluator confidence on Q1	3	2
Q2: The objectives of the service are met	4	4
Evaluator confidence on Q2	3	3
Q3: The service is novel to my knowledge	4	4
Evaluator confidence on Q3	3	3
Q4: I am satisfied with the performance of the service	4	4
Evaluator confidence on Q4	3	3
Q5: Overall, I am satisfied with this service	4	4
Evaluator confidence on Q5	3	3

With respect to the acceptability of the Dicode Collaboration Support services, the evaluators overall agreed that the services have all the functionality they expected (median = 4, mode = 3), the interface of the services are pleasant (median = 4, mode = 4) and that they will recommend these services to their peers/community (median = 4, mode = 3).

The analysis of qualitative evaluation results showed that, overall, reviewers found the services ‘promising’, ‘easy and intuitive’, as well as ‘very useful for a complex use case’. However, a few technical and documentation issues were raised, such as: ‘A bit slow loading time both for the workspace list and the mind-map view’; ‘The arrows’ graphics were not very pleasant for me: they start from the middle of the icon and not from the beginning of the square... the overall idea however, is quite good’; ‘I got a bit confused until I fully understand what I had to do’; ‘I often missed some system information’.

Usability measures assessed for the Dicode Collaboration Support services included (Table 3): tolerance, physical mapping, conceptual models, feedback, error prevention, flexibility, ease of recognition, flexibility of the use efficiency, provision of clear error messages, aesthetics of the minimalist design, help and documentation facilities, user control capabilities, as well as consistency and presentation standards. As shown, the lowest mean values were 5.53 and 5.88, corresponding to the rating of the ‘help and documentation facilities’ and the ‘user control capabilities and freedom of action’, respectively (the scale in this case was 0–10). In line with some qualitative evaluation results reported above, such findings reveal the need for more detailed documentation of the services, as well as for provision of help files and system messages. Finally, as shown in Table 3, the highest reported mean values with the lowest variability concerned the services’ physical mapping, conceptual models, and consistency and presentation standards.

**Table 3:** Usability Principles Descriptive Statistics for the Dicode Collaboration Support Service.

Question	min	max	mean	sd
Rate the tolerance: behaviour similar to expectations	2	10	6.71	1.929
Rate the physical mapping: conceptual correspondence between commands and functions	3	10	6.94	1.952
Rate the conceptual models: the operation of the proposed actions according to the perception of user for these actions	3	10	6.88	1.764
Rate the feedback: notification regarding the user's position	3	9	6.12	1.764
Rate the error prevention: restrict user errors & support for their solution	4	10	6.06	1.676
Rate the flexibility: variety of operation modes	2	9	6.33	2.056
Rate the ease of recognition: easy identification of the required actions	2	10	6.02	2.243
Rate the flexibility of the use efficiency: shortcuts provision, configuration capabilities	2	10	6.71	2.176
Rate the provision of clear error messages: simple language in error messages and a proposal to resolve them	4	10	6.71	1.649
Rate the aesthetics of the minimalist design: messages with the necessary information	1	10	6.35	2.370
Rate the help facilities and the documentation facilities: help facilities related to user action	2	9	5.35	2.095
Rate the user control capabilities and the freedom of action: understandable and direct processes as undo and redo	2	9	5.88	2.342
Rate the consistency and presentation standards: maintain the same presentation of the interface	3	10	7.53	2.035

## Discussion and Conclusions

As shown in the previous sections, the Dicode platform (i.e. the Dicode Workbench and integrated Dicode services) is a user-friendly tool that exploits the synergy between human and machine reasoning to facilitate and enhance data-intensive and cognitively-complex collaboration. Integrated at the core of the platform, the Dicode Collaboration Support services handle the aggregation of different users' perspectives. In addition, the Dicode platform is able to augment the quality of collaborative research (users may save time by skipping unnecessary tasks, accomplishing trivial tasks faster, while the platform provides a remedy to the information and cognitive overload).

Collaboration between users can be easily enhanced through the meaningful integration of independently developed approaches and datasets. Users may easily customize the Dicode Workbench through a proper assembly of web services and associated data resources that suit to their needs. Through an integrated registry of services, users may be informed about the functionality of each service available (in any case, the selection of the appropriate service and/or dataset can be facilitated through the exchange of ideas and arguments within a Dicode workspace). At the same time, the platform may exploit third-party web applications, which are often tailored to the evolving needs of various research communities. For example, data analysis applications can be uploaded provided that they are wrapped in a web service. This offers a great opportunity for researchers to upload their own code and collaborate with their peers in improving it, or discussing their findings by using one of the above mentioned Dicode services. Moreover, the proposed solution is appropriate when new volumes of data are incrementally incorporated to update the outcome of a certain method, as well as when provenance of data and certain workflow decisions need to be retained.

During the data analysis process, the platform enables users to set up a highly interactive process, where they can easily decide about which data repositories should be considered, trigger and parameterize the associated data mining mechanisms, explore their discovery patterns (possibly using descriptive summary statistics), discuss the weaknesses of the identified patterns, control the output's complexity, and set up new iterations of the data mining algorithm by defining other descriptive statistics or considering alternative data. It is in our future plans to develop services that will provide on-line access to R console through the Dicode Workbench. This would considerably increase Dicode's flexibility, for instance, in analyzing raw data since quality control protocols or services for data normalization would be easily included.

From a web evolution perspective, Dicode provides a single web-based infrastructure that is flexible enough to accommodate heterogeneous tasks, such as data mining and collaborative sense making, which are typically handled by separate systems. This alleviates expenses related to the large-scale data loading into multiple systems. Equally important, the development of the Dicode platform has followed a component-based approach, based on open standards and custom web technology; this allows an easy extension of the platform by using and adapting existing resources (i.e. data resources and data analysis tools), or developing new ones to cover the needs of related contexts.

The proposed solution allows for new working practices that may convert information overload and cognitive complexity to a benefit of knowledge discovery. This is achieved through properly structured data that can be used as the basis for more informed decisions. Simply put, the Dicode solution is able to turn information growth into knowledge growth; it improves the quality of the outcome of a collaboration process, while enabling users to be more productive and focus on creative activities.
